# Complexes of Oligoribonucleotides with *D*-Mannitol Inhibit Hemagglutinin–Glycan Interaction and Suppress Influenza A Virus H1N1 (A/FM/1/47) Infectivity In Vitro

**DOI:** 10.3390/ph10030071

**Published:** 2017-08-09

**Authors:** Nataliia Melnichuk, Larisa Semernikova, Zenoviy Tkachuk

**Affiliations:** Institute of Molecular Biology and Genetics, National Academy of Sciences of Ukraine, 03680 Kyiv, Ukraine; natalia.melnichuk8@gmail.com (N.M.); larisasemernikova@gmail.com (L.S.)

**Keywords:** hemagglutinin, influenza virus, oligoribonucleotides-d-mannitol complexes

## Abstract

The influenza virus hemagglutinin (HA) mediates both receptor (glycan) binding and membrane fusion for cell entry and has been the basis for subtyping influenza viruses. The oligoribonucleotides-d-mannitol (ORNs-d-M) complexes possess an anti-influenza activity in vitro and in vivo. In the present studies, we have found that ORNs-d-M interferes with hemagglutinin (HA)–glycan interaction and suppress viral infection in host cells. HA–glycan interactions were evaluated to indirectly quantify the amount of influenza virus titer by an agglutination assay. Influenza virus infectivity was determined by TCID_50_ assay. The direct virucidal action of the complexes was evaluated by both cytopathic effects (CPE) reduction assay and cell MTT assay. We found that ORNs-d-M hinders interaction between HA and glycan. These complexes decreased the infectivity of influenza virus and had a direct virucidal action. ORNs-d-M reduces influenza virus infectivity, affecting the HA–glycan interaction in vitro. By suppressing the influenza viral infection, the ORNs-d-M can have direct virucidal action.

## 1. Introduction

Influenza virus belongs to the *Orthomyxoviridae* family and consists of four types: A, B, C, and D. Among these types, the influenza A viruses causes major infections in respiratory tract of humans and lower mammals, and in gastrointestinal tract of birds, making it responsible for numerous deaths and great economic losses every year. Occasionally, the influenza A virus causes pandemics. The influenza virus contains two major surface glycoproteins: hemagglutinin (HA) and neuraminidase (NA). HA mediates both receptor (glycan) binding and membrane fusion for the cell entry, and NA functions as the receptor-destroying enzyme during the virus release. The influenza A viruses are classified into 16 HA (H1–H16) and 9 NA (N1–N9) sub-types of which H1–H3 have successfully adapted to humans [1,2]. Both HA and NA are important antigens that determine antigenic variation of the influenza viruses and host immunity. Variability in HA is primarily responsible for the continual evolution of new strains and subsequent influenza epidemics [3].

Aptamers are single-stranded DNA (ssDNA) or RNA sequences that can bind with high affinity and specificity to a wide range of target molecules such as proteins, cell surface receptors, and even whole cells [4,5,6] as well as other organic or inorganic molecules such as ATP, dyes, amino acids, drugs, or simple small cations [7]. Theoretically, aptamers can be used therapeutically in any disease for which extracellular blocking of protein–protein interactions is required [2,8,9]. 2′-5′-Oligoadenylates (2′-5′-As) can bind to some proteins (interferon-α, S100 calcium-binding protein A1) of signaling pathways of innate immunity with relatively weak (micromolar) dissociation constant and change a conformation of these proteins [10,11].

Natural oligoribonucleotides (ORNs) (total yeast RNA) have a wide range of biological activities and can be used in antiviral treatment, playing a key role in antiviral activity [12]. It was shown in a study that oligoribonucleotides-d-mannitol (ORNs-d-M) complexes (total yeast RNA modified with D-mannitol (D-M)) possess antiviral activity against the influenza A (H1N1), avian influenza, and parainfluenza viruses [13]. It was also shown that, with the influenza A (H1N1) infection of MDCK, the maximum tolerated concentration of the ORNs-d-M was 5.0 mg/mL, the minimal active concentration was 31 μg/mL, while its chemotherapeutic index was 161 [14]. An effective dose of the ORNs-d-M for prevention of the influenza A (H1N1) infection in vitro is 1.25–10 mg/mL and 0.6–10 mg/mL for treatment. The ORNs-d-M intraperitoneal and intravenous injections to mice for the prevention in doses from 15 to 150 mg/kg have high anti-influenza activity. Intranasal administration of the ORNs-d-M for prevention in these same doses demonstrated anti-influenza activity 10-times higher compared to the intraperitoneal and intravenous injections [14]. However, the mechanisms of the ORNs-d-M anti-influenza activity are still not clear.

In this research, we found that the ORNs-d-M efficiently interfere with HA–glycan interactions according to the agglutination assay. The ORNs-d-M not only inhibit the HA–glycan interactions, but also reduces influenza A (H1N1) virus infectivity. It was demonstrated that the ORNs-d-M have direct virucidal action on the influenza A virus H1N1 (A/FM/1/47).

## 2. Results

### 2.1. Interaction of the Influenza Virus A H1N1 HA with Glycan and Inhibition of This Interaction by the ORNs-d-M

**Figure 1:**
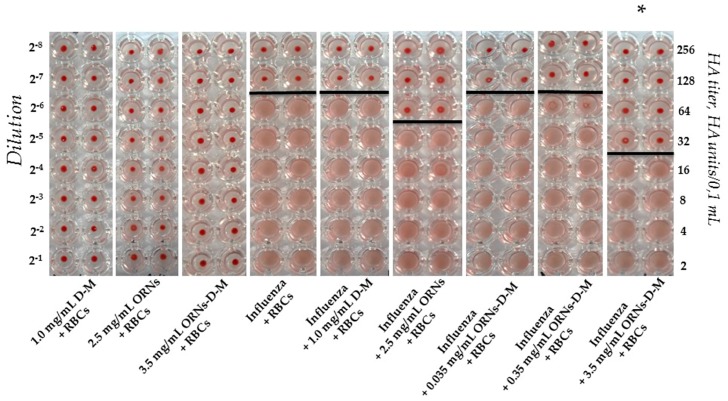
The ORNs-d-M inhibition of the human RBCs 0 (І) agglutination mediated by the influenza A virus H1N1 (A/FM/1/47) HA proteins. D-M + RBCs: negative control of the D-M (1.0 mg/mL); ORNs + RBCs: negative control of the ORNs (2.5 mg/mL); ORNs-d-M + RBCs: negative control of the ORNs-d-M (3.5 mg/mL); Influenza + RBCs: influenza positive control; Influenza + D-M + RBCs: test of the influenza preincubated with 1.0 mg/mL of the D-M; Influenza + ORNs + RBCs: test of the influenza preincubated with 2.5 mg/mL of the ORNs; Influenza + ORNs-d-M + RBCs: test of the influenza preincubated with the ORNs-d-M at concentrations of 3.5, 0.35, and 0.035 mg/mL. The HA titer was tested by HA assay. The documented photo is shown as one of eight independent experiments. Statistical significance was evaluated using the Student’s *t*-test, relative to the influenza positive control (* *p* < 0.05).

The agglutination assays have commonly been exploited to indirectly quantify the titer of the influenza virus, utilizing the specific interaction of the HA of the influenza virus with glycans expressed on the cell surface [15]. As shown in Figure 1, a view of the hemagglutination reaction mixtures displayed in a round-bottomed microwell plate after the addition and dilution of the ORNs, D-M, and ORNs-d-M as negative controls. Hemagglutination of red blood cells (RBCs) was not observed in the wells containing the ORNs, D-M, and ORNs-d-M. The RBCs agglutinated by the influenza virus were maintained in a suspended distribution and looked as a diffuse reddish solution. Based on this hemagglutination analysis the HA titers were calculated as the reciprocal of the minimum dilution forming the red button, indicating one HA unit. The RBCs agglutination by the influenza virus preincubated with the 2.5 mg/mL of ORNs and 3.5 mg/mL of ORNs-d-M was decreased compared with the influenza control, whereas agglutination of the RBCs by influenza virus preincubated with the 1.0 mg/mL of D-M and 0.35 and 0.035 mg/mL of ORNs-d-M remained unchanged. Thus it was shown that the ORNs-d-M at concentration 3.5 mg/mL decreased the HA titer of influenza virus by four times after the incubation of virus with this drug in comparing to the virus control (*p* < 0.05) (Figure 1).

### 2.2. Decrease of the Influenza Virus Infectivity after Incubation with the ORNs-d-M

**Figure 2:**
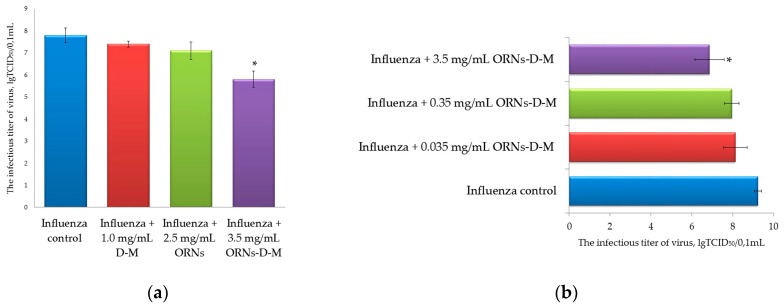
(**a**) Decrease of the influenza A virus H1N1 (A/FM/1/47) infectious titer after incubation with the ORNs-d-M; (**b**) Effects of different concentrations of the ORNs-d-M on infectious titer of the influenza A virus H1N1 (A/FM/1/47). Influenza control: virus without drug preincubation, Influenza + D-M: virus preincubated with 1.0 mg/mL of the D-M; Influenza + ORNs: virus preincubated with 2.5 mg/mL of the ORNs; Influenza + ORNs-d-M: virus preincubated with 3.5, 0.35, and 0.035 mg/mL of the ORNs-d-M. The infectious titer of influenza virus was determined by the Reed–Muench method (TCID_50_ assay). Data are shown as the mean ± SD for eight independent experiments. Statistical significance was evaluated using the Student’s *t*-test, relative to the control of influenza virus (* *p* < 0.05).

After 48 h incubation of MDCK cells with the influenza virus that had been previously incubated with the D-M, ORNs, and ORNs-d-M at 20 °C for 30 min, the infectious titers of these viruses were determined by TCID_50_ assay [16]. This analysis allowed us to evaluate the effect of these drugs on the influenza virus infectivity. The infectious titer of the influenza virus preincubated with the 3.5 mg/mL of ORNs-d-M was significantly decreased by 2 lgTCID_50_ (*p* < 0.05) in comparison to the virus control (Figure 2a,b). Conversely, the infectious titer of influenza virus after incubation of the virus with 2.5 mg/mL of ORNs, 1.0 mg/mL of D-M, and 0.035 and 0.35 mg/mL of ORNs-d-M decreased insignificantly in comparison to the virus control.

### 2.3. Direct Virucidal Action of the ORNs-d-M on the Influenza A Virus H1N1 (A/FM/1/47)

Apoptosis induced during the influenza virus infection is a major contributing factor to cell death and tissue damage [17,18]. The studies with the 1918 pandemic virus in macaques showed that the activation of the apoptotic pathway was a source of tissue damage during infection [19]. We hypothesized that the ORNs and ORNs-d-M have an inhibitory effect on binding of influenza A virus to MDCK cells.

MDCK cells were infected with the influenza virus (infectious titer in cell culture—lg 4, HA titer—1:128 HAU) that had been previously incubated with the ORNs and ORNs-d-M. After 48 h of incubation to facilitate viral infection into the host cells, the virus-induced cytopathic effects (CPEs) were observed under a light microscope and the cell viability was measured using the MTT reagent. The viability of cells infected with the influenza virus was significantly decreased by 49% (*p* < 0.05) compared to control cells. It was also detected that the viability of cells, infected with influenza virus preincubated with 2.5 mg/mL of the ORNs and 3.5 mg/mL of the ORNs-d-M, was significantly higher by 38% and 43% respectively (*p* < 0.05) in comparison to the control influenza virus.

**Figure 3:**
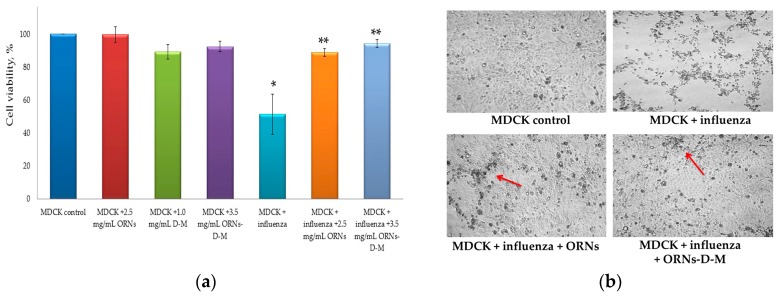

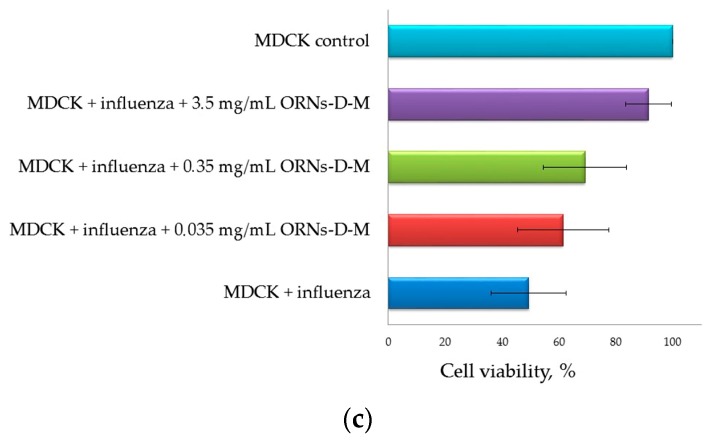
(**a**) Increased viability of MDCK cells infected influenza A virus H1N1 (A/FM/1/47) owing to the ORNs and ORNs-d-M; (**b**) Inhibition of the influenza virus -induced CPEs by the ORNs and ORNs-d-M in MDCK cells; (**c**) Increased viability of the cells infected with the influenza virus preincubated with the ORNs-d-M was in a dose-dependent manner. MDCK control: normal cells without influenza and drugs; MDCK + ORNs: cells incubated with 2.5 mg/mL of the ORNs; MDCK + D-M: cells incubated with 1.0 mg/mL of the D-M; MDCK + ORNs-d-M: cells incubated with 3.5 mg/mL of the ORNs-d-M; MDCK + influenza: cells infected with influenza virus without drug preincubation; MDCK + influenza + ORNs: cells infected with influenza virus preincubated with 2.5 mg/mL of the ORNs; MDCK + influenza + ORNs-d-M: cells infected with influenza virus preincubated with 3.5, 0.35, and 0.035 mg/mL of the ORNs-d-M. The cell viability was tested by MTT assay and the virus-induced CPEs were observed under a light microscope. Data are shown as the mean ± SD for five independent experiments. Statistical significance was evaluated using the Student’s *t*-test, relative to the cell control (* *p* < 0.05) and to the virus control (** *p* < 0.05).

The MDCK viability was insignificantly decreased by 10% during infection with virus preincubated with the ORNs compared with the control of the ORNs, whereas at the influenza + ORNs-d-M infection the MDCK viability remained unchanged compared to the ORNs-d-M control (Figure 3a).

Additionally, after 48 h, monolayers were examined for CPEs by using a light microscope. As shown in Figure 3b, the prominent and moderate CPEs were observed in the cells infected with the control influenza virus, whereas CPEs were minor in the cells infected with the influenza virus that had been incubated with the ORNs and ORNs-d-M (the red arrow indicates minor CPEs). The enhanced viability of the influenza infected cells was observed depending on the concentration of the ORNs-d-M used for incubation with the influenza virus (Figure 3c).

## 3. Discussion

Currently, the main influenza interventions are the annual trivalent or quadrivalent vaccines (who.int/influenza/vaccines), but because of rapid antigenic drift and shift in influenza viruses, selection of appropriate vaccine strains is a formidable task (cdc.gov/flu/about/season/vaccine-selection.htm) [20,21,22,23]. Furthermore, the small-molecule therapeutic space against influenza virus is currently limited to four licensed drugs: neuraminidase inhibitors oseltamivir (Tamiflu) and zanamivir (Relenza), which prevent release of nascent virions [24], and amantadine (Symmetrel) and rimantadine (Flumadine), which are M2 ion channel inhibitors [25,26]. However, the emergence of drug-resistant influenza variants has led to a decline in the efficacy of these drugs [26,27,28]. Therefore, new influenza therapeutics with novel mechanisms of action and against new targets are urgently required to combat the persistent threat of influenza viruses. Moreover, co-infection with influenza and other respiratory viruses decreases efficiency of the some aforementioned drugs [29,30]. So it is important to search for the influenza antiviral drugs with a wide spectrum of antiviral action.

As it was mentioned above, aptamers are single-stranded DNA (ssDNA) or RNA sequences that can bind with high affinity and specificity to a wide range of target molecules, such as proteins, cell surface receptors, and even whole cells [4,5,6]. 2′-5′-Oligoadenylates (2′-5′-As) have a wide spectrum of antiviral action. They affect proteins of 2′-5′-oligoadenylate synthetase (OAS)/RNase L pathway and this manner lead to the destruction of viral RNA [31]. The 2′-5′-As can bind to the proteins of signaling pathways of innate immunity such as interferon-α, S100 calcium-binding protein A1 (S100A1) with relatively weak (micromolar) dissociation constant and change conformation of these proteins by affecting their secondary structures [10,11].

Recently, it has been found that the complexes of oligoribonucleotides-d-mannitol based on total yeast RNA have antiviral activity against DNA and RNA viruses with a wide spectrum of antiviral action [12]. It was shown that 1.0–3.0 mg of the ORNs-d-M inhibited neuraminidase activity of influenza virus by 50% [14]. 

In the process of infection, the influenza virus initially requires binding of the HA protein to its cell surface receptor glycan, followed by membrane fusion. In the presented study, the ORNs-d-M were evaluated for their ability to interfere with the HA–glycan interactions using the agglutination assay. The ORNs-d-M was found to have an inhibitory effect on the binding of the influenza virus HA proteins to the sialic acid receptors of the RBCs. For example, the ORNs-d-M at a concentration of 3.5 mg/mL decreased the HA titer of influenza virus by four times (Figure 1). Next, we estimated an ability of the ORNs-d-M to affect influenza A virus H1N1 infectivity and detected that 3.5 mg/mL of the ORNs-d-M decreased the infectious titer of influenza virus (Figure 2). These results suggest that the ORNs-d-M can recognize the viral HA, likely at or around the receptor binding region required for the penetration of the influenza virus into host cells [32]. Our results also suggest that the ORNs-d-M potentially can bind to amino acids near the glycan binding region [2], change the conformation of protein [11], and prevent efficient HA–glycan interaction. To validate the direct virucidal action of the ORNs-d-M in blocking viral binding and entry into the host cells, we evaluated the ability of these complexes to influence cell viability and CPE reduction during the influenza infection. A dose of 3.5 mg/mL of the ORNs-d-M were shown to enhance by 94% the cell viability and to reduce CPEs during influenza infection (Figure 3a–c). The results obtained suggest that the ORNs-d-M can have direct virucidal action on the influenza A virus H1N1 by blocking the HA–glycan interactions and inhibiting neuraminidase activity [14].

Our results show that one of mechanisms of the ORNs-d-M anti-influenza activity is direct virucidal action by blocking HA–glycan interactions and inhibiting neuraminidase activity of influenza virus [14]. We believe that innate immunity induction by ORNs-d-M under influenza infection can be by other mechanisms responsible for anti-influenza activity of these complexes. In future research, the binding the ORNs-d-M with HA of influenza virus should be studied to understand the mechanism of inhibiting the HA–glycan interaction more precisely. Also, the effects of ORNs-d-M on expression of innate immunity genes should be studied under influenza infection.

## 4. Materials and Methods

### 4.1. Materials

Oligoribonucleotides (ORNs)—total yeast RNA with the dominant fraction of 3–8 nucleotides. ORNs-d-Mannitol (ORNs-d-M) complexes—total yeast RNA is modified with D-mannitol (D-M) in mixing ratio 2.5:1.0 mg. The ORNs and ORNs-d-M were purchased from Goodwill Associates, Inc. (Boca Raton, FL, USA).

Madin–Darby canine kidney (MDCK) cells were obtained from Russian Cell Culture Collection and influenza virus A/FM/1/47 (H1N1) were obtained from National Virus Collection of D.I. Ivanovsky Institute of Virology of Russian Academy of Medical Science (Moscow, Russia).

MDCK cells were maintained in our laboratory in RPMI (SIGMA, USA) supplemented with 10% fetal bovine serum (FBS) (SIGMA, St. Louis, MO, USA), antibiotic antimycotic solution (SIGMA, Rehovot, Israel), and HEPES (SIGMA, St. Louis, MO, USA).

### 4.2. Methods

#### 4.2.1. Agglutination Assay

To evaluate the ability of the ORNs-d-M complexes to inhibit agglutination of erythrocytes by the influenza A virus H1N1 (A/FM/1/47) we conducted the hemagglutination assay. The assays were performed on a round-bottomed 96-well plate using 1% human red blood cells 0 (І) (RBCs) in phosphate-buffered saline (PBS). Fifty microliters of PBS buffer (pH 7.4) were added to each well. The influenza virus was incubated with ORNs (2.5 mg/mL), ORNs-d-M (3.5 mg/mL), and D-M (1 mg/mL) (SIGMA, St. Louis, MO, USA) at 20 °C for 30 min. In the first well of column 50 µL of control or test sample were added. Each well sample was mixed and 50 µL were transferred to the next well on its right. Mixing was repeated and 50 µL were transferred down the length of the plate. The 50 µL from the last well was discarded. To each well, 50 µL of 1% RBCs working solution were added and mixed gently. The reaction mixtures were incubated at room temperature for 60 min to allow agglutination, followed by photography to document the results. Influenza positive control was virus incubated without drugs at 20 °C for 30 min. Negative control of the ORNs, ORNs-d-M, and D-M were conducted under similar conditions but without influenza virus. The drugs were dissolved in PBS. The negative results appeared as dots in the center of round-bottomed plates. The positive results formed a uniform reddish color across the well. The virus’ HA titer was determined as the number of the highest dilution factor that produced a positive reading.

#### 4.2.2. Infectious Titer of Influenza Virus (TCID_50_ Assay)

Decrease of the influenza virus infectious titer after the incubation with RNA drugs was determined using the TCID_50_ assay by the method of Reed–Muench. The infectious titer of the influenza virus was evaluated by the infection of MDCK cells and hence the virus titer was expressed as the tissue culture infective doses leading to 50% infected cells (TCID50) [16]. MDCK cells were seeded on 4 × 96-well plates (Orange Scientific, Braine-l’Alleud, Belgium) (5 × 10^3^/well) in RPMI with 10% FBS. After 24 h of incubation, growth medium was removed from each well of 4 × 96-well plate and the cells were washed three times with 35 µL/well of RPMI containing 2 µg/mL trypsin TPCK (SIGMA, USA). The influenza virus was divided into four test tubes: influenza control (virus preincubated without drugs), influenza + ORNs (virus preincubated with 2.5 mg/mL of the ORNs), influenza + D-M (virus preincubated with 1.0 mg/mL of the D-M), influenza + ORNs-d-M (virus preincubated with 0.035, 0.35, and 3.5 mg/mL of the ORNs-d-M) and incubated at 20 °C for 30 min. Then we diluted each of the preincubated influenza from 10^0^ to 10^-10^. Each of 4 × 96-well plates with the cells was infected with one preincubated virus (e.g., one plate infected with different dilutions of the “influenza + D-M”) adding 35 µL/well of the influenza virus (eight wells were infected with one dilution of virus). The control cells in each 96-well plate (eight wells without influenza virus infection) were added 100 µL of serum-free RPMI containing 2 µg/mL of trypsin TPCK. Then we allowed virus to adsorb to cells at 37 °C for 45 min. After incubation, the virus was removed, discarded from all wells, and the 100 µL of RPMI containing 2 µg/mL of trypsin TPCK without FBS was added to each well and the cells were further incubated at 37 °C for 48 h. The control cells (without influenza virus infection) were maintained as-is. After 48 h of incubation, the infectious titer of each of the viruses (influenza control; influenza + ORNs; influenza + D-M; influenza + ORNs-d-M) was expressed as the tissue culture infective doses leading to 50% infected cells.

#### 4.2.3. Direct Virucidal Action of the ORNs-d-M (MTT and CPE Reduction Assays)

Twenty-four hours before the experiment, MDCK cells were seeded on 96-well plate (5 × 10^3^/well) in RPMI with 10% FBS. After incubation, growth medium was removed from each well and the cells were washed three times with 35 µL/well of RPMI containing 2 µg/mL trypsin TPCK and were infected at 37 °C for 45 min with 35 µL/well of the influenza A virus (the cell culture infectious titer—lg 4.0, HA titer—1:128 HAU). The influenza A virus had been incubated at 20 °C for 30 min with the ORNs and ORNs-d-M at concentrations of 2.5 mg/mL and 3.5, 0.35, and 0.035 mg/mL respectively. Virus preincubation was carried out like during analysis of the influenza virus infectious titer (TCID_50_ assay). Negative control (influenza control) was cells infected with influenza virus and without drugs. Positive control (MDCK control) was cells incubated without influenza viral and these drugs. After 45 min of incubation, 35 µL of simple solution was removed and discarded from all wells. The 100 microlitrs of serum-free RPMI containing trypsin TPCK (2 µg/mL) was added to each well and the cells were further incubated at 37 °C for 48 h. For control drugs, the cells that after 24 h incubation in RPMI with 10% FBS were incubated in 100 µL drugs/serum-free RPMI (2.5 mg/mL of the ORNs, 1 mg/mL of the D-M, 3.5 mg/mL of the ORNs-d-M) at the same conditions as other cells for 48 h. The virus-induced CPEs were observed under a light microscope. Viability of the cells was determined by the MTT assay after 48 h of incubation. Serum-free RPMI was removed from all wells and discarded and 100 µL/well of MTT buffer (15 µL of 5 mg/mL the 3-(4,5-dimethylthiazol-2-yl)-2,5-diphenyltetrazolium bromide (MTT) (SIGMA, St. Louis, MO, USA) and 85 µL of serum-free RPMI) were added. The cells in MTT buffer were further incubated at 37 °C for 4 h and then MTT buffer was removed and discarded from all wells and 150 µL of DMSO was added to each well. Optical density was measured by an EL × 800 Absorbance Reader (BioTek, Winooski, VT, USA) at a 570 nm wavelength. The data were expressed as % of viability cells related to 100% of controls. 

#### 4.2.4. Statistical Analysis

The data were presented as mean ± SD. A two-way analysis of Student’s *t*-test was used to analyze the data for agglutination, TCID_50_, and MTT assays. For all the tests, *p* ≤ 0.05 was considered significant.
